# Self-Care in Patients With Inflammatory Bowel Disease: A Descriptive Cross-Sectional Multicenter Study

**DOI:** 10.1093/crocol/otaf061

**Published:** 2025-10-24

**Authors:** Daniele Napolitano, Silvia Cilluffo, Valeria Amatucci, Davide Bartoli, Valentina Biagioli, Piergiorgio Martella, Alessandro Monaci, Antonello Cocchieri, Ercole Vellone

**Affiliations:** Department of Biomedicine and Prevention, Tor Vergata University, Rome, 00133, Italy; CEMAD-Fondazione Policlinico Gemelli IRCCS, Rome, Italy; Department of Biomedical Sciences for Health, University of Milan, Milan, Italy; CEMAD-Fondazione Policlinico Gemelli IRCCS, Rome, Italy; Unit of Anesthesia, Intensive Care and Pain Medicine, Sant’Andrea University Hospital, Rome 00189, Italy; Department of Medical and Surgical Sciences—DIMEC, University of Bologna, Bologna, Italy; Department of Biomedicine and Prevention, Tor Vergata University, Rome, 00133, Italy; Department of Biomedicine and Prevention, Tor Vergata University, Rome, 00133, Italy; Università Cattolica del Sacro Cuore, Rome, Italy; Department of Biomedicine and Prevention, Tor Vergata University, Rome, 00133, Italy; Faculty of Nursing and Midwifery, Wroclaw Medical University, Wrocław, Poland

**Keywords:** inflammatory bowel disease, self-care, ulcerative colitis, Crohn’s disease, patient education

## Abstract

**Introudction:**

Inflammatory bowel disease (IBD), encompassing Crohn’s disease (CD) and ulcerative colitis (UC), requires complex self-care behaviors to manage symptoms and maintain quality of life. Despite its importance, self-care in IBD remains poorly understood. This study aims to investigate self-care practices and the sociodemographic and clinical determinants of self-care among patients with IBD.

**Methods:**

A multicenter cross-sectional study was conducted in nine IBD units in Italy. Patients were enrolled between April and June 2024. Self-care was assessed using the Self-Care of Chronic Illness Inventory, covering self-care maintenance, self-care monitoring, and self-care management. Socio-demographic and clinical data were collected through structured questionnaires. Multiple linear regressions examined the relationships between patient characteristics and self-care dimensions. The N-ECCO Research Grant supported the study.

**Results:**

Among 452 patients (49.3% CD, 50.7% UC), mean self-care scores were 72.84 ± 12.57 (self-care maintenance), 81.14 ± 17.94 (self-care monitoring), and 67.73 ± 16.99 (self-care management). Ulcerative colitis patients demonstrated significantly better self-care management than CD patients (*P* = .002). Higher disease activity was associated with worse self-care maintenance (*β* = –.11, *P* = .030), while supplement use predicted better self-care maintenance (*β *= .10, *P* = .028). For self-care monitoring, female gender (*β *= .11, *P* = .020) and supplement use (*β *= .13, *P* = .005) were positively associated with higher scores.

**Conclusion:**

Inflammatory bowel disease patients demonstrated adequate self-care maintenance and monitoring, but their self-care management was suboptimal. Female gender and supplement use were associated with better self-care monitoring; disease activity worsened self-care maintenance. Ulcerative colitis patients had better self-care management than CD, highlighting the need for tailored interventions to improve self-care.

## Graphical Abstract

**Graphical Abstract:**
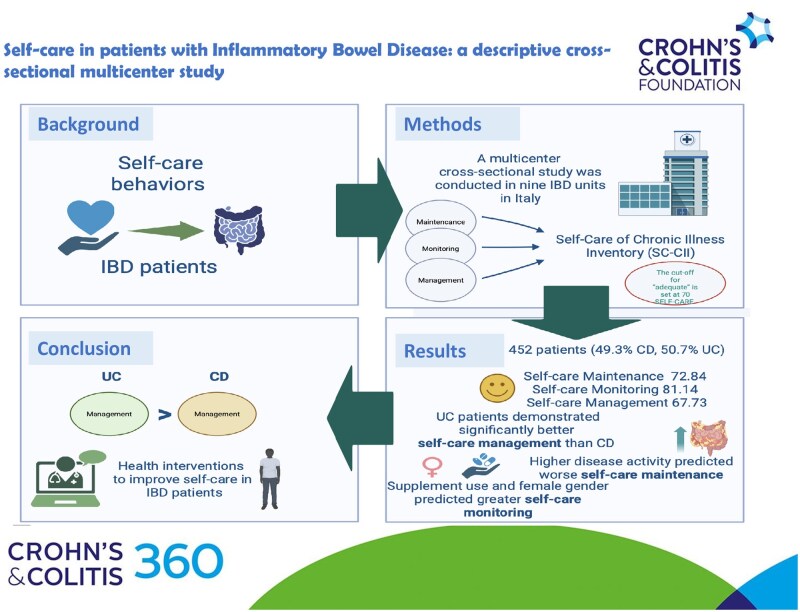

Key Messages

- **What is already known?** 
  Self-care is a critical component in the management of chronic diseases, including inflammatory bowel disease (IBD). Still, existing studies have mainly focused on qualitative findings or specific aspects such as treatment adherence, without using validated instruments.
- **What is new here?** 
  This is the first multicenter study to quantitatively assess self-care behaviors in IBD patients using the Self-Care of Chronic Illness Inventory. It identifies significant differences in self-care dimensions between patients with Crohn’s disease (CD) and ulcerative colitis. It highlights the role of gender, disease activity, and supplement use in shaping self-care behaviors.
- **How can this study help patient care?** 
  By revealing key socio-demographic and clinical predictors of self-care, this study supports the development of tailored interventions to improve self-care, especially among patients with CD and those with low education or high disease activity.

## Introduction

Inflammatory bowel disease, which includes ulcerative colitis (UC) and Crohn’s disease (CD), consists of chronic inflammatory conditions of the gastrointestinal tract, marked by episodes of symptom exacerbation and remission.^1^^,^^2^ Approximately, the 0.5% of the global population is affected by IBD, and this prevalence is expected to rise to 1% by 2030, potentially affecting over 10 million individuals in the Western world.^3^

Many patients with IBD experience insufficient disease control with treatments, which results in complications and disability.^4^ The unpredictable nature of IBD, combined with the requirement for complex self-care practices, such as adhering to medication regimens, monitoring symptoms, managing diet and stress, and making timely decisions in response to symptom changes, leads to the need for regular patient-provider interactions.^5^ Self-care is essential for IBD patients as it enhances symptom control, prevents flare-ups, and improves overall quality of life.^6^ This requires a better understanding of IBD patient self-care practices and the elements which affect their self-care activities.

According to the Middle-Range Theory of Self-Care of Chronic Illness, self-care is defined as the “process of maintaining health through health-promoting practices and managing illness.”^7^ This theory has three main components: self-care maintenance (ie, behaviors to maintain the disease stable), self-care monitoring (ie, actions adopted to monitor the disease signs and symptoms), and self-care management (ie, behavioral response to signs and symptoms of exacerbations). Even though the theory was developed for all chronic conditions, it also adapts very well to IBD.^8^ Inflammatory bowel disease patients should undertake specific behaviors to maintain disease stability, such as taking medications as prescribed and attending routine medical appointments; to monitoring IBD signs and symptoms, for example, keeping track of stool frequency and consistency; and, to manage symptoms as they occur, such as contacting a healthcare provider in response to flare indicators like increased stool frequency, blood in the stool, or persistent abdominal pain.

While several studies have been conducted in other chronic conditions (eg, heart failure, pulmonary diseases, diabetes), self-care research in IBD remains limited despite the importance of self-care for these patients.^9–12^ Two qualitative studies have explored self-care behaviors in IBD patients^13^^,^^14^ and both have reported behaviors aligned with the self-care management and monitoring domains. In fact, Mohsenizadeh et al. described how individuals develops coping strategies, adapt to social and work environments, and strengthen self-efficacy when facing disease-related challenges. At the same time, Lovén Wickman et al. found that participants actively monitor symptoms with diaries, and identify patterns linking flares to diet and stress. Other studies^15–17^ have used a quantitative approach to study self-care and were focused on self-care management behaviors such as the response to IBD exacerbations and proactive decision-making through symptom monitoring. These studies suggest that people with IBD engage in self-care behaviors.^13^^,^^14^

Self-care research in IBD is also limited in the identification of sociodemographic and clinical factors influencing self-care. Age and time since diagnosis are key determinants of better self-care and that younger patients and those with a recent diagnosis of IBD experience greater challenges in managing their condition. At the same time, individuals with a more extended disease history develop more effective self-care strategies.^17^ However, these studies were conducted in a single country, which may limit the generalizability of the results to other healthcare contexts.^17^ Also employment status was found to influence self-care, as individuals who are employed often face time limitations and competing responsibilities that can hinder engagement in self-care activities.^18^

Additionally, while educational attainment and family support are generally associated with better self-care (eg, through higher education levels and strong social networks that facilitate improved disease management), existing research does not fully account for potential confounding variables such as socioeconomic status and access to healthcare resources.^13^^,^^16^ The research also lacks a thorough investigation of gender-related aspects. Research on other chronic diseases shows that male diabetic patients participate more in physical activity. At the same time, women focus primarily on symptom tracking, even though but their glycemic control remains less effective.^19^

Regarding IBD self-care, Mizuno et al. found key differences in self-care strategies between CD and UC based on disease management levels. Due to the disease’s variability, CD patients tend to develop stronger self-care skills, including dietary awareness and symptom monitoring based on biomarkers. In contrast, UC patients tend to follow more stable, long-term management routines, such as regularly adhering to maintenance therapy and attending clinical check-ups and follow-up.^17^ Unlike other chronic conditions where disease severity often hinders self-care, in IBD, it seems that self-care is shaped more by disease characteristics and monitoring needs than by severity alone.^10^^,^^12^

Given the unpredictable nature of IBD, a comprehensive understanding of self-care maintenance, self-care monitoring, and self-care management in a more systematic and theoretically-based way is essential to improve patient outcomes. Recent findings suggest that self-care behaviors are associated with improved quality of life and lower disease activity in patients with IBD^20^; however, the literature on self-care and the impact of socio-demographic and clinical factors remains sparse. Existing studies provide limited and sometimes conflicting evidence.

## Material and Methods

### Aims

This study aimed to measure the frequency and level of self-care behaviors in patients with IBD and to explore their associations with sociodemographic and clinical characteristics.

### Design and settings

We used a descriptive cross-sectional design. Participants were recruited from 9 IBD outpatient units across Italy, including both public hospital settings and accredited private centers. These units provided specialized care for patients with IBD and were selected to ensure geographic and institutional diversity. Patients were recruited during outpatient visits between April 2024 and June 2024.^21^

### Procedures

Eligible patients were identified during routine outpatient visits at the participating IBD centers. Recruitment occurred on pre-specified days, and both physicians and trained research staff informed patients about the study. Patients were approached in the clinic waiting rooms or at the end of their medical appointments. All participants received a written patient information sheet and were allowed to ask questions—those who agreed to participate provided written informed consent before completing the questionnaire.

### Sample

A convenience sample was collected from consecutive patients diagnosed with IBD (CD and UC). We asked participants to complete a self-report questionnaire either independently or through a face-to-face interview after signing the informed consent form. Inclusion criteria for participation included being 18 year or older, having a confirmed diagnosis of IBD, and providing a voluntary agreement to participate. The exclusion criteria were a diagnosis of IBD for less than 12 months, surgery within the last 6 months, and the presence of another severe chronic disease. These criteria were applied to ensure that participants had experienced a sufficient period of disease stability and adaptation, allowing for the assessment of established self-care behaviors rather than those influenced by recent diagnosis or postoperative recovery.^22^

### Socio-demographic and clinical questionnaire

The research team developed a comprehensive questionnaire to collect socio-demographic data, habits, and clinical characteristics. This was designed to gather detailed information on age, gender, education, and occupation as well as clinical factors, such as type and duration of IBD (in years), disease activity, and current therapy (eg, biologic, immunosuppressive, or steroid therapy). The survey also recorded information on previous surgeries and occurrences of perianal disease. Disease activity was assessed using a combination of clinical symptoms, biochemical markers, and endoscopic findings in both UC and CD patients. For UC, the partial Mayo score was used; for CD, the Harvey-Bradshaw Index. Biochemical remission was defined as CRP ≤ 0.5 mg dL^−1^ and fecal calprotectin (FCP) ≤ 250 μg/g. Endoscopic disease activity was assessed using the Mayo endoscopic subscore for UC and the SES-CD for CD24.^23-25^ Data were collected by IBD specialists during the patient’s visit or extracted from clinical records assessed within the past 60 days. Based on available information, disease activity was classified into four levels: remission, mild, moderate, and severe. For analytical purposes, these four categories were collapsed into two groups: remission–mild and moderate–severe, to improve statistical power and interpretability in the results.

### Self-Care of Chronic Illness Inventory

The Self-Care of Chronic Illness Inventory (SC-CII)^26^ was used to assess self-care. This theory-based instrument measures the frequency of self-care behaviors across three scales: self-care maintenance (5 items), self-care monitoring (6 items), and self-care management (7 items). Each item is rated on a 5-point Likert scale ranging from 1 (“never”) to 5 (“always”), with standardized scores from 0 to 100. Higher scores indicate greater engagement in self-care behaviors. Item 13 (“Monitor for symptoms”) belongs to the self-care monitoring scale. Still, it has been conceptually defined as a bridge item between self-care monitoring and self-care management, as it represents a pivotal step linking the observation of symptoms to the decision-making process for symptom response.^27^ Unlike other items, Item 13 uses a distinct Likert scale assessing the timeliness of symptom recognition and response.

The SC-CII demonstrated good psychometric performance in a recent Italian validation study among IBD patients (*N* = 456), showing excellent model fit in confirmatory factor analysis (Comparative Fit Index - CFI = 0.978, Tucker-Lewis Index - TLI = 0.966, Root Mean Square Error of Approximation - RMSEA = 0.054, Standardized Root Mean Square Residual - SRMR = 0.032). Internal consistency was high across all scales (McDonald’s ω: 0.83-0.88), and construct validity was supported by moderate-to-strong correlations with self-care self-efficacy.^8^

### Data analysis

Descriptive statistics were used to calculate means, standard deviations, percentages of the variables, and correlations among socio-demographic characteristics and the three self-care scales. An independent samples *t*-test compared self-care item responses between patients with CD and UC. Missing data were assessed at both the item and individual levels. Kendall’s Tau-b correlation coefficients were calculated for associations involving ordinal categorical variables (eg, education level) and continuous variables (eg, self-care monitoring scores), as this method appropriately measures the strength and direction of monotonic relationships between such variable types.

Multiple linear regression analysis was performed using SPSS version 29 to address the research question. Socio-demographic characteristics were regressed separately on self-care maintenance, self-care monitoring, and self-care management scales as performed in other studies using the same instrument.^28^^,^^29^ Statistical significance was set at *P* < .05. Normality of study variables was tested with the Kolmogorov–Smirnov test (*P* > .05).

### Ethical considerations

The study was conducted in accordance with Good Clinical Practice and the Revised Declaration of Helsinki. The Territorial Ethics Committee (Lazio 3) reviewed and approved the protocol (Approval No. 0023486/23, dated August 2, 2023). Before enrollment, all participants received detailed oral and written information regarding the study’s objectives and procedures and provided written informed consent.

### Funding

This publication is the first result of the awarded N-ECCO Grant 2024.

## Results

### Descriptive data

A total of 456 patients were enrolled and gave the signed informed consent form (Table 1). Of these, 452 (99.1%) completed the questionnaire in full and were included in the analysis, but four were excluded because gave incomplete responses to the core items of the SC-CII. Participants included almost an equal number of CD (49.3%) and UC (50.7%) patients, with a mean age of 43.5 years (SD 16.4). The gender distribution was balanced, with 50.9% male and 49.1% female. Most had a high school (44.7%) or university education (32.5%), and 63.3% was student. The 57.3% was in remission, 42.7% had active disease, and 11.9% was in severe condition. Most patients (71.9%) received biological therapy, and 14.2% had a perianal disease. Thirty-eight percent of patients had undergone at least one surgery (Table 1).

**Table 1.** Socio-demographic and clinical characteristics.

	Total	CD	UC	*P-value*
Total	452	223 (49.3%)	229 (50.7%)	
Gender. *N* (%)				*.29*
Male	230 (50.9%)	120 (26.5%)	103 (22.9%)	
Female	222 (49.1%)	110 (24.3%)	119 (26.3%)	
Age. Mean (SD)				*.72*
Years	43.46 (16.4)	43.24 (16.5)	43.68 (16.3)	
Education. *N* (%)				*.27*
Primary education	15 (3.3%)	6 (1.3%)	9 (2.0%)	
Secondary education	49 (19.5%)	39 (8.6%)	49 (10.8%)	
High school	93 (44.7%)	109 (24.1%)	93 (20.6%)	
University degree	147 (32.5%)	69 (15.3%)	223 (49.3%)	
Occupation. *N* (%)				*.85*
Worker	251(55.5%)	130 (28.8%)	121 (26.8%)	
Non-workers	201 (44.5%)	100 (22.1%)	101 (22.3%)	
Disease activity. *n* (%)				***.00024***
Remission–mild	328 (72.6%)	196 (43.4%)	132 (29.2%)	
Moderate–severe	124 (27.4%)	27 (6%)	97 (21.5%)	
Current therapy. *n* (%)				*.004*
Biological therapy	325 (71.9%)	175 (38.7%)	150 (33.2%)	
Other therapies	127 (28.1%)	52 (11.5%)	75 (16.6%)	
Use supplements				*.37*
Yes	269 (59.5%)	95 (21.0%)	88 (19.5%)	
No	183 (40.5%)	128 (28.3%)	141 (31.2%)	
Perianal disease. *n* (%)				***.0001***
No	388 (85.8%)	177 (39.2%)	211 (46.7%)	
Yes	64 (14.2%)	46 (10.2%)	18 (4.0%)	
Years since diagnosis. Mean (SD)				*.29*
Years	11.6 (9.4)	11.6 (10.1)	11.72 (8.7)	
Previous surgeries. *n* (%)				***.00001***
Never	313 (69.2%)	111 (24.6%)	202 (44.7%)	
1	65 (14.4%)	55 (12.2%)	10 (2.2%)	
More 1	74 (16.4%)	57 (12.6%)	17 (3.8%)	
Urgent care in the last 12 months. *n* (%)				*.12*
No	305 (67.5%)	162 (35.8%)	143 (31.6%)	
Yes	147 (32.5%)	61 (13.5%)	86 (19.1%)	

Abbreviations: CD, Crohn’s disease; UC, Ulcerative Coltis. Bold values indicate statistical significance at *P* < 0.01.

Chi-square test was used for categorical variables; Independent samples *t*-test was used for continuous variables.

### Self-care behaviors

The overall levels of the self-care revealed mean scores of 72.84 ± 12.57 for self-care maintenance, 81.14 ± 17.94 for self-care monitoring, and 67.73 ± 16.99 for self-care management scale. The mean scores for self-care maintenance and self-care monitoring did not differ significantly between patients with CD and UC. Conversely, self-care management showed a statistically significant difference between the groups (*P* = .002), with UC patients scoring higher (70.23 ± 16.97) than those with CD (65.17 ± 16.68) (Table 2).

**Table 2.** Self-Care of Chronic Illness Inventory (SC-CII) item means for total sample, CD and UC subgroups.

		Total mean (SD)	CD mean (SD)	UC mean (SD)	*P*-value
	Self-care maintenance	72.84 (12.57)	73.56 (11.70)	72.14 (13.36)	.233
	Item				
1	Make sure to get enough sleep?	3.66 (1.01)	3.66 (1.03)	3.66 (1.00)	ns
2	Try to avoid getting sick	4.18 (0.98)	4.20 (0.95)	4.15 (1.00)	ns
3	Do physical activity	3.51 (1.17)	3.49 (1.14)	3.53 (1.20)	ns
4	Eat a special diet	3.71 (1.03)	3.76 (1.05)	3.66 (1.02)	ns
5	See your healthcare provider for routine health care	4.87 (0.42)	4.87 (0.41)	4.87 (0.43)	ns
6	Take prescribed medicines without missing a dose	4.71 (0.63)	4.77 (0.61)	4.65 (0.65)	ns
7	Manage stress	2.76 (1.32)	2.84 (1.28)	2.69 (1.36)	ns
	*Self-care Monitoring*	81.14 (17.94)	80.00 (18.62)	82.25 (17.23	.183
	Item				
8	Monitor your condition	4.35 (0.73)	4.29 (0.76)	4.41 (0.70)	ns
9	Monitor for medication side-effects	4.14 (1.06)	4.09 (1.10)	4.20 (1.02)	ns
10	Pay attention to changes in how you feel	4.30 (0.90)	4.31 (0.87)	4.28 (0.92)	ns
11	Monitor whether you tire more than usual doing normal activities	3.98 (1.03)	3.95 (1.05)	4.01 (1.00)	ns
12	Monitor your condition	4.46 (0.83)	4.36 (0.91)	4.55 (0.73)	.01
	*Self-care Management*	67.73 (16.99)	65.17 (16.68)	70.23 (16.97)	.002
	Item				
14	Change what you eat or drink to make the symptom decrease or go away	4.14 (1.02)	4.11 (1.03)	4.17 (1.02)	ns
15	Change your activity level (eg, slow down, rest)	3.46 (1.17)	3.51 (1.15)	3.41 (1.19)	ns
16	Take a medicine to make the symptom decrease or go away?	3.54 (1.34)	3.33 (1.32)	3.74 (1.32)	.001
17	Tell your healthcare provider about the symptom at the next office visit	4.34 (1.00)	4.24 (1.06)	4.44 (0.92)	.03
18	Call your healthcare provider for guidance	3.69 (1.30)	3.50 (1.32)	3.88 (1.25)	.001
19	Think of a treatment you used the last time you had symptoms. Did the treatment you used make you feel better	2.75 (1.39)	2.58 (1.46)	2.92 (1.30)	0.01

Abbreviations: CD, Crohn’s Disease; UC, Ulcerative Colitis; ns, not significant (*P *> 0.05)

Differences between groups were assessed using independent samples *t*-tests.

**Figure 1.**
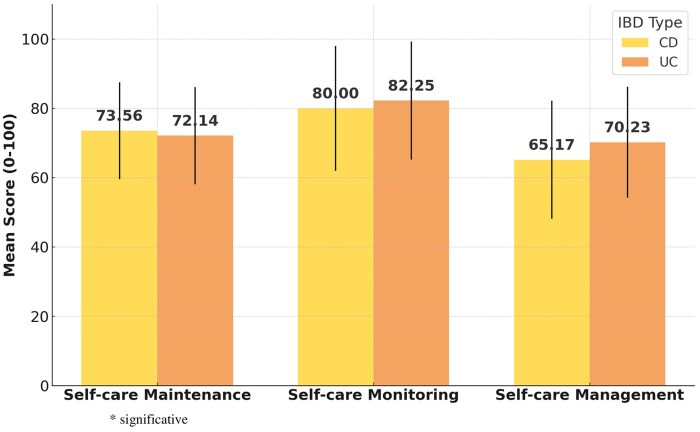
Mean scores of self-care dimensions for CD and UC.

Figure 1 compares self-care maintenance, self-care monitoring, and self-care management between the two groups.

The Tau Kendall correlation coefficients between the categorical and continuous independent variables and the three self-care scales appear in Table 2. The results indicate positive bivariate correlations between supplement use and all self-care scales’ scores (self-care maintenance: τ  =  0.100, *P *< .05; self-care monitoring: τ  =  0.129, *P *< .01; and self-care management: τ  =  0.109, *P *< .01). Self-care maintenance scores had negative associations with disease activity (τ = –0.099, *P *< .01) and occupation (τ = –0.093, *P *< .05), but self-care monitoring had a positive association with the female gender (τ = –0.112, *P *< .01). Self-care management had a negative association with previous surgery (τ = –0.079, *P *< .05).

### Item-level analysis of SC-CII

The analysis of individual SC-CII items is shown for each construct in Table 3. In self-care maintenance, the behaviors which were more often performed included attending routine healthcare appointments (4.87 ± 0.43) and taking prescribed medications without missing doses (4.71 ± 0.63), while the lowest scores were for managing stress (2.76 ± 1.32) and physical activity (3.51 ± 1.17).

**Table 3.** Correlation of socio-demographic characteristics and self-care.

Dimensions	Gender	Education	Occupation	Disease activity	Type of pathology	Current therapy	Using supplements	Perianal disease	Previous surgery	Age	Years of diagnosis	SCMaint	SCMon	SCMan
Gender	1													
Education	0.28	1												
Occupation	**−0.124^a^**	**0.224^a^**	1											
Disease activiy	0.49	−0.033	−0.028	1										
Type of pathology	0.058	−0.010	0.010	**0.371^a^**	1									
Current therapy	0.020	−0.048	0.020	−0.038	−0.004	1								
Supplements use	0.091	**0.132^a^**	−0.045	0.016	−0.043	0.037	1							
Perianal disease	0.007	0.025	−0.033	0.007	**−0.183^a^**	−0.025	0.040	1						
Previous surgery	**−0.118^b^**	−0.036	−0.049	−0.077	**−0.416^a^**	−0.040	0.085	**0.293^a^**	1					
Age	0.015	−**0.232^a^**	−**0.081^b^**	−0.034	0.013	−0.020	0.015	0.007	**0.078^b^**	1				
Years of diagnosis	−0.007	−0.055	**0.081^b^**	−0.060	0.038	0.013	−0.019	0.057	**0.146^a^**	**.245^a^**	1			
SCMaint	−0.004	0.072	−**0.093^b^**	−**0.099^a^**	−0.050	0.054	.100**^b^**	0.019	0.020	−0.048	−0.061	1		
SCMon	−**0.112^a^**	0.049	−0.019	0.031	0.051	0.078	**0.129^a^**	0.054	−0.004	0.017	0.026	**0.269^a^**	1	
SCMan	0.044	0.033	−0.036	0.027	**0.0125^a^**	0.013	**0.109^a^**	0.026	−**0.079^b^**	0.008	0.041	**0.245^a^**	**0.292^a^**	1

Abbreviations: SCMaint, Self-care maintenance; SCMon, Self-care monitoring; SCMan, Self-care management.

a *P < *.01.

b *P < *.05. We calculate Tau Kendall correlation coefficients among the categorical and continuous independent variables.

**Table 4.** Multiple linear regression models of socio-demographic characteristics on self-care behaviors.

Self-care maintenance	B	SE	β	t	95% CI of B	P-value	R^2^
Variables							0.047
Gender	0.019	1.20	.001	0.016	−2.35 to 2.38	.98	
Age	−0.02	0.04	−.03	−0.50	−0.102 to .061	.62	
Education	1.21	0.79	.08	1.53	−0.34 to 2.76	.13	
Occupation	0.07	0.65	.005	0.110	−1.21 to 1.34	.91	
Type of pathology	0.26	1.39	.01	0.19	−2.47 to 2.99	.85	
Disease activity	−1.29	0.59	−.11	−2.17	−2.46 to −0.122	**.03^a^**	
Current therapy	3.07	2.20	.065	1.39	−1.256to 7.398	.16	
Supplements use	2.69	1.22	.10	2.21	0.297 to 5.086	**.028^a^**	
Perianal disease	1.35	1.79	.04	00.75	−2.179 to 4.870	.45	
Years of diagnosis	−0.11	0.07	−.08	−1.524	−.24 to 0.031	.13	
Surgical previous	0.41	0.91	.02	0.46	−1.372 to 2.200	.65	
**Self-care monitoring**	**B**	**SE**	**β**	**t**	**95% CI of B**	***P*-value**	**R^2^**
Variables							0.053
Gender	3.99	1.72	.11	2.33	0.629-7.36	**.02^a^**	
Age	0.048	0.059	.044	0.82	−0.069 to 0.163	.42	
Education	1.20	1.12	.05	1.07	−1.004 to 3.404	.28	
Occupation	−0.26	0.921	−.014	−0.285	−2.072 to 1.547	.78	
Type of pathology	2.73	1.975	.076	1.383	−1.128 to 6.64	.17	
Disease activity	0.18	0.85	.011	0.22	−1.479 to 1.848	.83	
Current therapy	4.56	3.13	.07	1.45	−1.601 to 10.71	.15	
Supplements use	4.94	1.73	.13	2.85	1.530-8.343	**.005^b^**	
Perianal disease	2.93	2.55	.06	1.15	−2.08 to 7.95	.25	
Years of diagnosis	0.004	0.09	.002	00.042	−0.19 to 0.199	.97	
Surgical previous	0.47	1.29	.020	0.367	−2.067 to 3.016	.71	
**Self-care management**	**B**	**SE**	**β**	**t**	**95% CI of B**	***P*-value**	**R^2^**
Variables							0.057
Gender	1.13	1.62	.033	0.69	−2.057 to 4.322	.49	
Age	−0.030	0.056	−.029	−0.54	−0.140 to 0.080	.59	
Education	0.69	1.06	.03	0.65	−1.126 to 3.11	.52	
Occupation	0.506	0.871	.028	0.58	−1.205 to 2.22	.56	
Type of pathology	4.81	1.87	.14	2.57	1.131-8.496	**.011^a^**	
Disease activity	−0.07	0.80	−.004	−0.08	−1.65 to 1.51	.93	
Current therapy	1.63	2.96	.026	0.55	−4.191 to 7.452	.58	
Supplements use	4.56	1.64	.13	2.77	1.33-7.79	**0.006^b^**	
Perianal disease	3.75	2.41	.077	1.55	−0.993 to 8.49	.12	
Years of diagnosis	0.151	0.094	.084	1.61	−0.033 to 0.335	.33	
Surgical previous	−1.69	1.223	−.76	−1.389	−4.102 to 0.705	.16	

a *P* < 0.05.

b *P* <0.01.

In self-care monitoring, patients were most attentive to tracking their condition (4.46 ± 0.83) and noticing changes in their feelings (4.30 ± 0.90). Still, they were less likely to monitor whether they tired more than usual during daily activities (3.98 ± 1.03).

Regarding item 13 (evaluating symptom recognition and response), overall, 10.6% of patients reported never experiencing symptoms (CD: 12.1%, UC: 9.2%), while 9.7% did not recognize them as disease-related (CD: 11.7%, UC: 7.9%). Only 3.5% reported recognizing symptoms very slowly (UC: 5.2%, CD: 1.8%), whereas 25.9% recognized them quickly, more frequently in CD (30.9%) than UC (21.0%) (*P* = .0039).

The more frequent self-care management behavior was reporting symptoms to the doctor during consultations (4.34 ± 1.00), whereas the least frequent was evaluating past treatments’ effectiveness (2.75 ± 1.39).

### Predictors of self-care in IBD

The regression model for self-care maintenance was statistically significant [F(11, 440) = 1.98, *P* = .028; R^2^ = 0.047]. Higher disease activity was associated with lower self-care maintenance (β = –.11, *P* = .030), while supplement use was a positive predictor (β = .10, *P* = .028).

For self-care monitoring, the model was also significant [F(11, 440) = 2.23, *P* = .012; R^2^ = 0.053]. Female gender (β = .11, *P* = .020) and supplement use (β = .13, *P* = .005) were associated with higher self-care monitoring scores.

The regression for self-care management was significant as well [F(11, 439) = 2.39, *P* = .007; R^2^ = 0.057]. Ulcerative colitis diagnosis (β = .14, *P* = .011) and supplement use (β = .13, *P* = .007) were both associated with better self-care management. All analyses are presented in Table 4.

## Discussion

This study examined self-care behaviors in IBD patients and determined which demographic and clinical factors influenced self-care behaviors in this population. Our findings give important information about self-care maintenance, self-care monitoring and self-care management, and show which variables affect these behaviors. The study emphasizes the importance of self-care for patients living with IBD^10^^,^^30^ and builds on the riddle-range theory of self-care in chronic illnesses,^7^ offering a comprehensive assessment of self-care behaviors in a population never studied before with a psychometrically sound instrument.

The levels of self-care maintenance and monitoring were adequate, with mean scores of 72.84 and 81.14, respectively, while self-care management was lower (M = 67.73).^31–33^ On average, patients reported almost always attending their scheduled medical visits and taking prescribed medication without missing doses. This finding contrasts with previous studies, which have often highlighted low adherence to treatment plans.^34^^,^^35^ Stress management received the lowest score among self-care maintenance behaviors, indicating that managing stress is probably a challenge for this population or that they lack adequate support. We know from prior studies that stress can act as a trigger for disease activity and can contribute to symptomatic flare-ups,^36–38^ consequently interventions to reduce stress may represent a research priority in future studies. Similar patterns related to stress were observed in other chronic diseases, such as heart failure and type 2 diabetes, where stress management and emotional support were key to improve effective self-care.^10^ In patients with heart failure, cognitive-behavioral therapy was already shown to improve self-care and quality of life,^39^^,^^40^ but in IBD this type of intervention was never adopted. In patients with diabetes, emotional distress is linked to poor adherence and self-management, highlighting the role of psychosocial factors in sustaining health behaviors.^41^

Self-care maintenance was negatively associated with educational level, indicating that individuals with lower education may struggle to perform self-care. This is consistent with previous studies showing that lower educational attainment is linked to poorer health outcomes and more challenging disease trajectories.^42^^,^^43^

The self-care monitoring scale scores were the highest in this study, and no differences between the two disease groups were found. These findings suggest strong adherence to medical recommendations related to symptom monitoring. Moreover, the lowest score the patients had in evaluating whether they got tired more than usual may indicate that some of them did not fully recognize the impact of their condition on their energy levels. This aspect may require specific interventions in future studies. However, the highest score within the monitoring items may be a sign that patients are concerned about their conditions.^44^ The disease groups showed statistically significant differences in this item score because UC patients performed the self-care monitoring behaviors more frequently. Patients with UC seem to require close symptom tracking because of their perceived need to monitor their condition often. Patients with UC tend to monitor their symptoms more regularly because the condition produces both predictable and visible symptoms, including rectal bleeding and urgency. Patients use this behavior as an adaptive approach to detect flares before they occur to keep their disease under control and protecting their quality of life. Monitoring for medication side effects received also a lower scores, suggesting limited awareness or educational needs in this area.^45^ Recognition of fatigue during daily activities had the lowest score within the monitoring scale, indicating that patients might struggle to distinguish changes in this area,^46^^,^^47^ possibly because fatigue is one of the most reported IBD symptoms.^48^

The results of self-care monitoring revealed a significant relationship with gender since women obtained higher scores than men. Female patients appear to track symptoms more actively than men, possibly because they have better health awareness or stronger body awareness. This finding aligns with a review that observed that women with chronic conditions, including type 2 diabetes, engage more actively in self-monitoring behaviors. The authors attribute this to gendered caregiving norms, which may also apply to IBD populations, reinforcing the link between traditional caregiving roles and enhanced symptom awareness.^18^^,^^49^ Gender-related differences in self‑care monitoring and self‑care management are well-documented across chronic conditions. In type 2 diabetes, women are more likely to engage in blood‑glucose monitoring and structured self-care routines compared to men.^50^ Moreover, women also report higher levels of anxiety and concern regarding diabetes management, which may trigger self-care tasks.^51^

In contrast to the generally adequate levels of self-care maintenance and self-care monitoring, self-care management emerged as the most challenging domain, with mean scores falling below the adequacy threshold.^52^ This finding suggests that many individuals with IBD may struggle to respond effectively to symptom exacerbations or to make autonomous decisions in managing their illness. A significant difference between diagnostic groups was observed: patients with UC demonstrated more adequate self-care management than those with CD. Although these differences reached statistical significance, the actual difference in mean scores (4.4 vs 4.2 points) is small and may not represent a meaningful clinical difference in patient behavior. Therefore, these findings should be interpreted with caution, emphasizing overall trends rather than focusing on minor variations between groups.

The difference in self-care management scores between CD and UC patients may reflects differences in disease presentation. Ulcerative colitis patients typically follow a more predictable and visible clinical course, characterized by symptoms such as rectal bleeding and urgency, which facilitate timely recognition and response.^53^^,^^54^ By contrast, CD patients tend to present with a more heterogeneous and often insidious symptomatology, including fatigue, diffuse abdominal discomfort, and fluctuating bowel habits, that may complicate symptom appraisal and delay both self-management and help-seeking.^4^^,^^55^ These subtler symptoms could reduce patients’ confidence in interpreting bodily cues, thereby undermining self-efficacy which is the most important factor in influencing self-care.^52^

Looking more closely at the specific management behaviors, patients were more likely to engage in reactive strategies, such as reporting symptoms or adjusting their diet, than in reflective or evaluative actions, such as reviewing the effectiveness of adopted treatments.^4^ This pattern may indicate a limited ability to critically appraise disease progression and treatment responses, underscoring the need for more structured education and empowerment interventions. Similar difficulties in symptom response are observed in other chronic diseases. In heart failure, patients often recognize early signs of deterioration but delay taking action due to low self-efficacy or uncertainty.^56^

In IBD, effective self-care management extends beyond symptom reaction—it requires reflective learning, informed decision-making, and treatment appraisal. These skills can be supported through targeted interventions, including nurse-led coaching, personalized care planning, and structured self-management programs, all of which have shown positive effects on patient self-efficacy and health outcomes.^54–58^ Empowering patients to assess their response to therapies and take appropriate action is essential for long-term disease control and quality of life.^59^ These findings highlight the value of tailored self-care strategies, particularly for patients with CD, who often face greater variability and complexity in symptom presentation. Interventions supported by IBD nurses or tracking tools may help bridge this gap, offering real-time feedback and structured guidance for decision-making.^54^^,^^57^ Similar approaches have proven effective in other chronic conditions. In hypertension, nurse‑led telehealth interventions improve blood pressure control and adherence.^60^ In type 2 diabetes, integrative technologies in nurse‑led interventions and mHealth tools (eg, telemonitoring, apps, phone follow‑ups) significantly enhance glycemic control, self-monitoring, and patient autonomy.^61^

Finally, while many patients in this study reported dietary adjustments to manage symptoms, this aligns with existing literature showing that patients frequently modify their diets despite limited scientific evidence linking specific nutritional patterns to disease control. Several reviews emphasize the lack of robust causal associations, suggesting a persistent gap between patient perception and the current scientific consensus.^54^^,^^62^^,^^63^ This underscores the need for more evidence-based and individualized nutritional counseling as part of routine clinical care.^64–66^

Our finding that supplement use correlates positively with self-care management is supported by the literature, which highlights that nutritional supplementation, particularly with iron and vitamin D, is often part of proactive disease management in IBD. This suggests that patients engaged in supplementation may also demonstrate higher health engagement overall.^67^ Supplements are used as beneficial tools to enhance nutritional health for IBD patients with documented deficiencies in iron, vitamins D, and B12. The administration of supplements needs clinical guidance because unregulated use can result in adverse effects and conflicts with pharmaceutical treatments.^68^ Medical professionals need to evaluate patient needs while tracking biochemical markers to deliver safe and precise supplementation. Similarly to our results, a study by Cushman et al. reported that surgical history in IBD patients, particularly in pediatric populations, is often associated with body image concerns and psychological distress. These factors may interfere with self-care engagement, highlighting the need for post-surgical support programs targeting self-efficacy and emotional adaptation.^69^ Surgical history in patients with IBD is often associated with changes in body image due to scars or stomas, which can lead to limitations in daily activities, mood disturbances such as anxiety and depression, and social withdrawal.^70^ These factors may negatively impact engagement in self-care, highlighting the need for comprehensive post-surgical support programs addressing both physical and psychological adaptation.

Patients with UC demonstrated greater engagement in self-care management behaviors compared to those with CD. They were more likely to take medication in response to symptoms, report issues to healthcare providers, and assess the effectiveness of previous treatments—indicating a more structured approach to symptom management. This may be explained by the more predictable and visible clinical course of UC, which facilitates symptom recognition and timely action. In contrast, the greater heterogeneity and variability of CD can complicate disease appraisal and self-care management, often requiring individualized care strategies. Crohn’s disease patients also tend to rely more on in-person healthcare services, which may reflect the increased complexity of their condition.^71^^,^^72^ These findings highlight the need to prioritize disease education and personalized support for CD patients to strengthen their self-care capacity and decision-making skills.

### Limitations and strengths

This study has some limitations that need to be recognised. First, because of its cross-sectional design, it is impossible to establish cause-and-effect relationships between socio-demographic or clinical factors and self-care behaviors. Second, while the multicenter approach increases the study’s external validity, the use of a convenience sample might introduce selection bias and reduce generalizability. Patients who voluntarily participated may be more engaged in their care or have better access to healthcare, which could lead to an overestimation of self-care behaviors. Third, self-care behaviors were measured by self-report tools that may be affected by recall bias or social desirability bias. For instance, patients may have over-reported adherence-related behaviors or underreported difficulties in stress management and fatigue recognition. This may limit the accuracy of specific self-care scores, especially those involving introspective or sensitive domains.

Furthermore, the relatively low variance explained by the regression models (between 0.5% and 0.6%) suggests that the socio-demographic and clinical factors considered in this study explain only a very small proportion of self-care behaviors. This result underlines the complexity of self-care, which is probably shaped by a variety of factors, including psychological factors (eg, resilience, self-efficacy), relational factors (eg, social support, patient-provider communication), and cultural factors (eg, health beliefs, cultural beliefs and attitudes to health and disease). This study included a heterogeneous group of IBD patients, including CD and UC, with a balanced gender distribution and adults of working age. It also includes patients with different levels of education, which helps to understand the role of education in disease management, as well as the need to tailor the support to those with lower levels of education. The geographic diversity of the sample provides insight into the differences in healthcare systems. In clinical practice, the study included the whole range of disease activity from remission to active disease, thus providing a wide range of results. The results confirm the need to develop individualized interventions for implementation into clinical practice.

This study provides a thorough and quantitative analysis of self-care activities among patients with IBD, distinguishing between self-care maintenance, self-care monitoring, and self-care management. One of the advantages of this study is the multicenter design that increases the external validity of the findings in other healthcare settings. Furthermore, the SC-CII was used to assess self-care in IBD patients, which is a validated and structured tool that closes an important gap in the literature. The results show that diverse socio-demographic and clinical characteristics of the patients influence self-care, which is important for developing effective interventions.

### Clinical practice implications

From a clinical perspective, these findings stress the need for targeted self-care education, particularly for CD patients with lower self-care management scores. Healthcare providers should develop individualized support plans that enhance patients’ ability to identify symptoms, take medication, and cope with stress. The observed differences between UC and CD patients suggest that disease-specific interventions may be required to address the specific issues related to each disease. Self-care education as part of standard IBD management may improve patient outcomes by encouraging patients to be more proactive in their disease monitoring and seeking medical care at the right time. The authors also recommend further research to explore the potential of tailored self-care interventions to enhance disease management and quality of life for patients with IBD.

## Conclusion

This study demonstrates that IBD patients exhibit different levels of self-care behaviors. Patients maintained proper self-care monitoring, but self-care management is challenging, especially for CD patients. The results show that patients with UC exhibited somewhat better self-care management compared to patients with CD, indicating differences in self-care behaviors between the two conditions. These findings highlight the need for individualized self-care interventions to enhance disease management. Healthcare providers should develop strategies to improve patient symptom detection, medication adherence, and active health monitoring, particularly for patients with IBD. Future studies should focus on developing interventions targeting self-care deficits and evaluating their impact on health outcomes.

## Data Availability

The data are available upon reasonable request from the author.
